# Characterization of HIV-1 subtype C envelope glycoproteins from perinatally infected children with different courses of disease

**DOI:** 10.1186/1742-4690-3-73

**Published:** 2006-10-20

**Authors:** Hong Zhang, Federico Hoffmann, Jun He, Xiang He, Chipepo Kankasa, John T West, Charles D Mitchell, Ruth M Ruprecht, Guillermo Orti, Charles Wood

**Affiliations:** 1 Nebraska Center for Virology, University of Nebraska, Lincoln, NE, USA; 2 School of Biological Sciences, University of Nebraska, Lincoln, NE, USA; 3 Department of Pediatrics, University Teaching Hospital, Lusaka, Zambia; 4 Department of Pediatrics, University of Miami School of Medicine, Miami, FL, USA; 5 Department of Cancer Immunology and AIDS, Dana-Farber Cancer Institute, Boston, MA, USA; 6 Department of Medicine, Harvard Medical School, Boston, MA, USA

## Abstract

**Background:**

The causal mechanisms of differential disease progression in HIV-1 infected children remain poorly defined, and much of the accumulated knowledge comes from studies of subtype B infected individuals. The applicability of such findings to other subtypes, such as subtype C, remains to be substantiated. In this study, we longitudinally characterized the evolution of the Env V1–V5 region from seven subtype C HIV-1 perinatally infected children with different clinical outcomes. We investigated the possible influence of viral genotype and humoral immune response on disease progression in infants.

**Results:**

Genetic analyses revealed that rapid progressors (infants that died in the first year of life) received and maintained a genetically homogeneous viral population throughout the disease course. In contrast, slow progressors (infants that remained clinically asymptomatic for up to four years) also exhibited low levels variation initially, but attained higher levels of diversity over time. Genetic assessment of variation, as indicated by dN/dS, showed that particular regions of Env undergo selective changes. Nevertheless, the magnitude and distribution of these changes did not segregate slow and rapid progressors. Longitudinal trends in Env V1–V5 length and the number of potential N-glycosylation sites varied among patients but also failed to discriminate between fast and slow progressors. Viral isolates from rapid progressors and slow progressors displayed no significant growth properties differences *in vitro*. The neutralizing activity in maternal and infant baseline plasma also varied in its effectiveness against the initial virus from the infants but did not differentiate rapid from slow progressors. Quantification of the neutralization susceptibility of the initial infant viral isolates to maternal baseline plasma indicated that both sensitive and resistant viruses were transmitted, irrespective of disease course. We showed that humoral immunity, whether passively acquired or developed *de novo *in the infected children, varied but was not predictive of disease progression.

**Conclusion:**

Our data suggest that neither genetic variation in *env*, or initial maternal neutralizing activity, or the level of passively acquired neutralizing antibody, or the level of the *de novo *neutralization response appear to be linked to differences in disease progression in subtype C HIV-1 infected children.

## Background

Mother to child transmission (MTCT) of human immunodeficiency virus type 1 (HIV-1) is the primary mode of pediatric HIV-1 infection [1] in sub-Saharan Africa. In this region, HIV-1 subtype C accounts for approximately 50% of infections. Pediatric HIV-1 disease progression has been most intensively studied for subtype B virus infections where it was found to be bimodal, with 15 to 20 % of untreated infants progressing rapidly to AIDS and death by 4 years of age [2], whereas the remaining 80% progress more slowly [3,4]. The applicability of such findings to other subtypes remains to be substantiated.

HIV-1 disease progression in adults is a complex interplay between viral factors, host genetics, and host immune response [5] where all contribute to disease progression [5-20]. The survival time for HIV-1 infected children is shorter, on average, than that of infected adults [21], and could be explained by a number of factors including: immaturity of their immune system [21], failure to acquire passive immunity from the mother, timing of transmission [2,22,23] or maternal HIV-1 RNA levels [24,25]. Other factors, such as viral replication rate, syncytium-induction, CD4^+ ^T-cell depletion, and thymic infection have been shown to associate with early onset of pediatric AIDS [25-28]. As in adults, the emergence of X4 variants in infected children has been associated with disease progression [27-29], but this is unlikely to be a causal factor since most rapidly progressing children harbor viruses of the R5 phenotype [21]. Moreover, shared HLA class I alleles between mother and infant was shown to influence clinical outcome [30]. Humoral immunity has been suggested to play a role in the disease for both adults and children, but the function of neutralizing antibody responses in delaying disease progression or preventing HIV-1 infection, especially in children, has not been fully established [5,19,20,31-33].

The determinants of many of the above biological properties map to the HIV-1 envelope glycoprotein (Env) or associate with Env receptor binding, tropism-definition, cytopathicity determinants or neutralization susceptibility [34-43], although other HIV-1 genes related to HIV-1 pathogenesis were also described [11,44-50]. Studies on HIV-1 Env from both infected adults and children have indicated that viral populations exhibiting high rates of non-synonymous nucleotide substitutions and high antigen diversity usually associate with broad immune reactivity, slow CD4^+ ^T cell decline, and slow rates of disease progression [33,51-54]. However, others have shown a correlation between higher sequence diversity and a more rapid disease onset [28,32]. Despite various associations with viral and host parameters, the mechanisms behind differential disease progression in HIV-1 infected children remain poorly defined.

As an extension of our efforts to better understand the characteristics of perinatally transmitted subtype C HIV-1 and to clarify the relationship between viral evolution, humoral immune responses and disease outcome in infected children [33], we analyzed the evolution of the *env *V1–V5 region from seven perinatally infected children with different disease courses. We also performed a longitudinal assessment of the infant neutralizing antibody responses against autologous primary viral isolates from various time points during disease progression. This study was designed to investigate the possible influence of genetic properties of subtype C envelope glycoproteins and humoral immune response on disease progression in infants.

## Results

### Characteristics of seven HIV-1 infected children

The subjects analyzed in this study were part of a mother/infant cohort followed for HIV-1 infection. Children were designated as rapid or slow progressors according to clinical assessment of outcome and time of survival. Infants 1449, 2669, 2873, and 2617 were considered rapid progressors since they died within the first year of life, due to apparent HIV-related complications. Slow progressors (infants 1984, 1084 and 1690) were followed for more than four years, and remained clinically asymptomatic for the duration of the study (Table 1). All children were anti-retroviral naïve throughout the study.

**Table 1:** Genetic variation, co-receptor usage and clinical information for the different infants included in this study

Sample	M	N	H	AA %	DNA %	dN/dS	PNGS	V1V5 length	Co-receptor usage	Cause of death
1449 i02m	2	26	21	0.6	0.3	0.71	21(18–21)	330(330-330)	CCR5	
1449 i04m	4	44	37	1.1	0.5	1.15	21(20–22)	330(323–330)		
1449 i08m	8	43	42	1.9	0.8	1.37	21(19–23)	328(327–330)	CCR5	Pneumonia
										
2669 i02m	2	30	29	1.1	0.6	0.61	25(23–25)	339(335–339)	CCR5	
2669 i06m	6	33	33	1.3	0.7	0.66	25(23–26)	337(329–339)	CCR5	Bronchitis
										
2873 i02m	2	29	26	1.2	0.6	0.91	28(27–29)	356(356-356)	CCR5	
2873 i04m	4	29	27	1.0	0.5	0.77	28(27–28)	356(356-356)	CCR5	Tuberculosis
										
2617 i02m	2	27	27	1.1	0.6	0.52	23(22–24)	336(336-336)		
2617 i04m	4	23	23	1.3	0.8	0.41	23(22–24)	336(336-336)		
2617 i06m	6	26	26	1.8	1.0	0.69	24(23–24)	336(336-336)	CCR5	Pyrexia
										
1984 i04m	4	25	25	2.1	1.0	0.97	21(19–22)	343(343-343)		
1984 i06m	6	27	26	1.7	0.7	1.21	22(20–24)	342(332–343)	CCR5	
1984 i12m	12	27	27	2.8	1.3	0.94	23(21–24)	340(331–343)	CCR5	
1984 i24m	24	26	26	4.2	2.0	0.98	24(22–26)	329(325–340)	CCR5	
1984 i36m	36	29	27	5.4	2.6	1.04	24(22–26)	332(326–341)	CCR5	
1984 i48m	48	26	26	6.6	3.3	1.02	24(22–26)	331(323–344)	CCR5	
										
1084 i06m	6	25	25	2.4	1.2	0.68	21(19–23)	319(319–328)		
1084 i27m	27	28	28	1.3	0.7	0.65	25(24–25)	328(328-328)	CCR5	
1084 i36m	36	25	24	5	2.6	1.1	25(22–28)	336(325–344)	CCR5	
1084 i48m	48	48	40	8.9	4.9	0.76	27(18–28)	344(325–344)	CCR5	
										
1690 i12m	12	28	28	1.8	1.0	0.71	26(23–27)	338(331–339)	CCR5	
1690 i24m	24	31	31	2.1	1.1	0.65	23(22–25)	329(325–336)		
1690 i36m	36	30	30	3.0	1.7	0.72	23(19–25)	327(319–338)		
1690 i48m	48	26	26	4.4	2.2	1.01	24(23–26)	336(331–341)	CXCR4/CCR5	

Months after birth (M); number of sequences per time point (N); number of unique haplotypes (H); mean number of pairwise amino acid differences as percentage (AA %); mean number of pairwise nucleotide differences as percentage (DNA %); ratio of non-synonymous (dN) to synonymous (dS) rate of substitution (dN/dS); number of putative N-linked glycosylation sites (PNGS) in Env V1–V5 region as median (min-max) and Env V1–V5 length in codons (V1V5 length) as median (min-max)

HIV-1 isolation was unsuccessful from all baseline (birth) samples and all infants were HIV PCR negative at birth, suggesting that they were infected either intrapartum or postpartum. HIV-1 *env *sequences were amplified from infant PBMC at different postpartum timepoints, as indicated in Table 1. Because the amount of sample from these children was limited, priority was given to virus isolation in lieu of PCR when necessary (e.g., infant 1084, viral isolation was positive by 4 month and the first PCR was performed 6 month after birth). A portion of the *env *gene from V1–V5 was amplified by PCR, cloned, and sequenced in order to longitudinally characterize Env genetic diversification and evolution.

### Env sequence analyses

We sequenced a total of 711 infant clones (23 – 48 sequences per timepoint) derived from PBMC genomic DNA. When all sequences were aligned and included in a single phylogenetic analysis, sequences from each mother-infant pair formed a monophyletic group, indicating that maternal and infant sequences were epidemiologically linked (data not shown). Viral subtype determinations showed that all cases were subtype C in Env, except for mother-infant-pair 1449 which was a subtype A/C recombinant.

**Figure 1:**
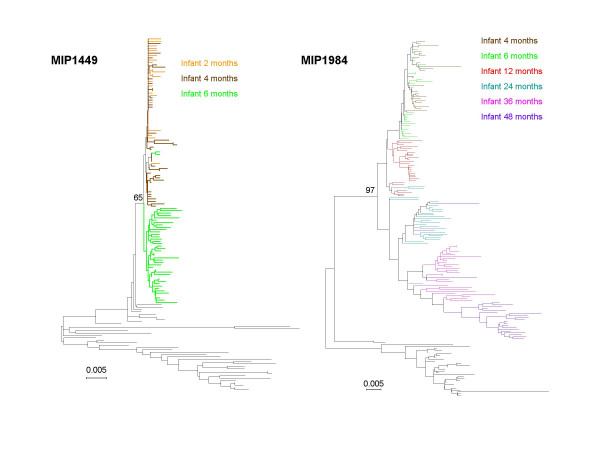
Neighbor-joining phylograms based on the Kimura 2 parameter genetic distance, showing relationships among infant sequences collected at different time-points, with a set of maternal sequences used for rooting purposes. Infant1449 is a rapid progressor, whereas infant 1984 corresponds to a slow progressor. Maternal sequences are in black in both cases, and branch colors correspond to the time of sample collection. Note that in both cases longer branches correspond mostly to sequences collected at later times. Bootstrap values are indicated at the nodes of the tree.

In all infants, the initial viral populations contained a reduced repertoire of *env *sequence variants when compared to the maternal population. These samples exhibited a large fraction of unique haplotypes, but with low nucleotide diversity, as would be expected in populations increasing in effective size from a limited set of founders (Table 1). Haplotype diversity (H/N in Table 1), an index of the number and relative frequency of unique sequences, ranged between 0.9 and 1.0, its maximum value, but average genetic distances within each sample remained low throughout the study (DNA % in Table 1). Mean genetic distance (DNA% in Table 1) were lower at the earliest time points, where they ranged from 0.3 to 1.2%, while for the latest, mean genetic distances ranged from 0.5 to 4.9 %. Representative phylogenetic analyses from a rapid progressor (1449) and a slow progressor (1984) are shown in Figure 1. Results from the different phylogenetic analyses for each mother-infant-pair were congruent among themselves, despite differences in the methods or weighting schemes used. In all cases, the results suggest that infections were established by highly homogeneous populations, with little phylogenetic structure among early sequences. In the case of fast-progressors, the diversity observed in different longitudinal samples taken from the infant was low relative to the mother, as indicated by the shorter branches leading to infant sequences when compared to the mother. A similar pattern can be observed for the earlier sequences of slow-progressors. Later time-point samples display longer branches in the phylogeny, as mutations accumulate and infant Env sequence diversity increases. It is important to note that trees from rapid and slow progressor were indistinguishable when analyses were restricted to sequences collected within 12 months after birth.

Similar patterns of variation were observed at the amino acid level, although levels of polymorphism were higher relative to variation at the nucleotide level. Mean amino acid differences (AA% in Table 1) within the initial populations ranged from 0.6 to 2.4 % for the earliest samples, and from 1.0 to 8.9% for the later time-point samples. Mean genetic distance (DNA % in Table 1) within contemporaneous sequences were lower in rapid progressors than in slow progressors (Table 1), but this difference was not statistically significant. There was a trend towards increased levels of genetic diversity as time progressed, with some refractory periods. Accordingly, we observed the highest levels of genetic diversity (DNA% in Table 1) in samples collected at the latest time points in slow progressors (Table 1, 48- month samples from infants 1084, 1690 and 1984). However, the rates of change in genetic diversity and genetic divergence were similar for all patients (data not shown), although sample sizes precluded statistical tests of this observation.

Positive Darwinian selection is indicated when the estimated ratio of non-synonymous changes to synonymous changes (dN/dS) >1. We observed high dN/dS values for the *env *gene (Table 1), suggesting that positive selection was occurring in the infant *env *genes. The values ranged from 0.41 to 1.37, with a mean of 0.78 (Table 1). The significance of this finding is that higher dN/dS values have been linked to longer survival, and presumably, a higher dN/dS value is a consequence of a stronger and/or broader immune response [20,55]. In the three slow progressor infants there was at least one time point where the dN/dS > 1; whereas a dN/dS > 1 was detected in only one of the rapid progressor infants (1449), but it is possible that this is a function of the duration of infection. Indeed, while we observed a higher level of non-synonymous substitutions in slow progressors (mean = 0.89) versus rapid progressors (mean = 0.78), this difference was not statistically significant.

**Figure 2:**
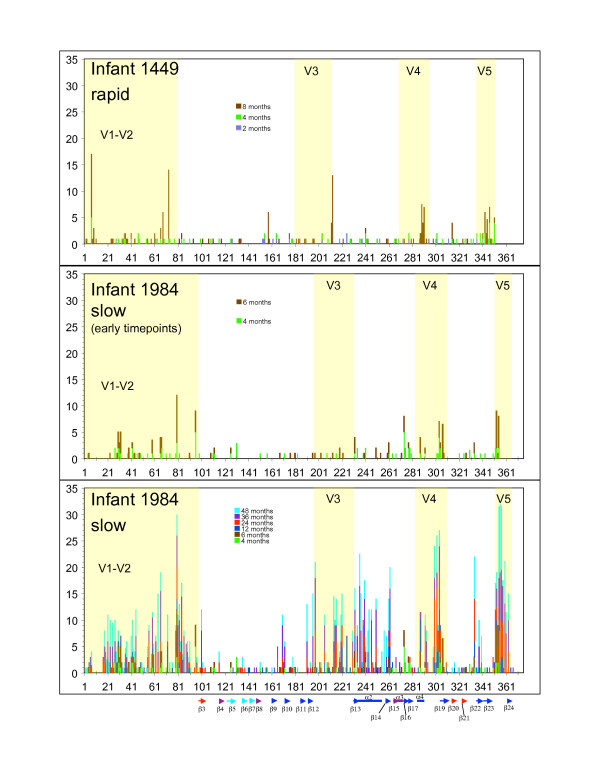
Estimated number of non-synonymous substitutions along the HIV-1 Env V1–V5 fragment sequenced, estimated in Datamonkey. Results are presented cumulatively for a rapid progressor (infant 1449) and a slow progressor (infant 1984), with the variable loops V1V2, V3, V4 and V5 shaded. The secondary structure elements (α helix and β sheet) are color coded as in Chen et al. [56].

To temporally and positionally visualize where non-synonymous changes occurred relative to 'constant' and 'variable' domains, as defined in subtype B, we compared the infant amino acid sequences to an alignment of HIV-1 HXB2 and the solved SIV glycoprotein structure by Chen et al. [56]. One representative infant from each group is shown in Figure 2. For clarity and ease of comparison to the rapid progressor infant 1449, we have separated the early time points from the complete analysis of slow progressor infant 1984. Inspection of the variation from both rapid and slow progressors revealed several common regions of the *env *sequence with high levels of non-synonymous variation and indicated that the C2 domain was the least variable, whereas the most variable areas were the V1–V2 loop, the 3' end of the C2 region, the V3 loop, the 5' end of C3, and the variable loops V4 and V5 (Figure 2). In addition, the variable loops V1–V2, V4 and V5 concentrated most of the indels observed. Comparison of 1449 and 1984 at similar time points (Figure 2, top and middle panels), revealed changes located in corresponding regions (e.g. V4 and V5), but there were also changes unique to either 1449 or 1984 (e.g. the 5' end of V1–V2 in 1449). Unfortunately, this study is not able to establish whether the unique mutations observed in 1449 are associated with rapid disease progression. There is also an accumulation of non-synonymous changes with time, particularly evident in 1984 where changes at many positions are cumulative, implying continued selection operating on positions over an extended time period (Figure 2, middle and lower panels). Whether this indicates immunological pressure or functional constraints for fitness remains to be determined. In contrast, for 1449 (Figure 2, top panel), a number of changes appear at only one time-point with no previous evidence of selection at that position.

Taken together, the higher diversity associated with later time points, in combination with the observed accumulation of amino acid substitutions in putatively exposed regions of the glycoprotein indicate that selective pressures, including humoral immunity, may be playing a substantial role in driving Env evolution.

### V1–V5 length and putative glycosylation sites

**Figure 3:**
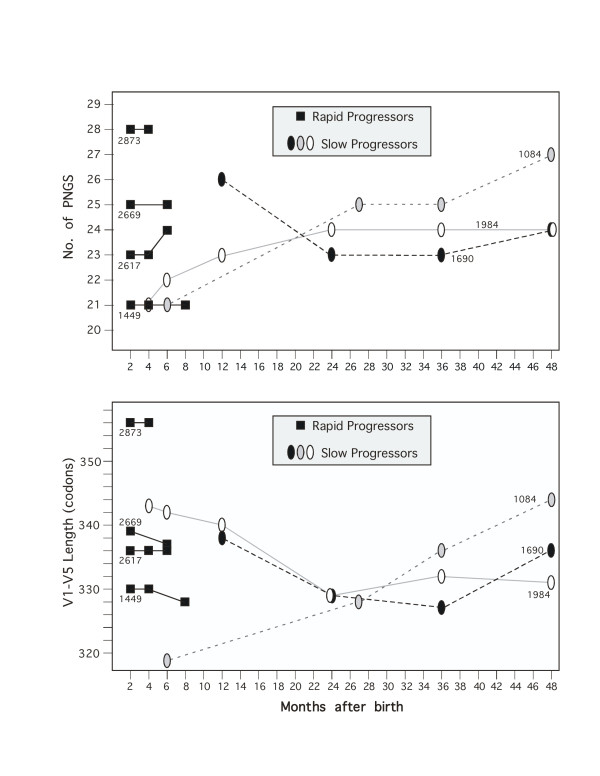
Longitudinal variation in the number of potential N-linked glycosylation sites (PNGS, top panel) and sequence length of the V1–V5 fragment sequenced (bottom panel) for both rapid progressors (1449, 2617, 2669 and 2873) and slow progressors (1084, 1690 and 1984).

The number of putative N-linked glycosylation sites (PNGS) and Env domain length have been hypothesized to modulate HIV-1 sensitivity to neutralization and to impact likelihood of transmission [57,58]. According to this hypothesis, shorter variants with fewer PNGS are expected in the earlier time-points, they have higher transmission fitness as the immune response of the recipient is still not developed; longer V1–V5 forms with more PNGS are expected to evolve at later time-points in response to increased and prolonged immune pressure. Longitudinal data including range and median values for Env V1–V5 length and PNGS are presented in Table 1, and the trend in median values in Figure 3. Rapid progressors exhibit a large range in both PNGS and V1–V5 length, with minor longitudinal changes during a period of up to 8 months postpartum. The number of PNGS is positively correlated with sequence length in these cases. Slow progressors show a tendency to increase (1984 and 1084) or decrease (1690) the number of PNGS with time, but the range of variation falls within the range observed for fast progressors (Figure 3, top panel). The same pattern is observed for longitudinal variation in V1–V5 length (Figure 3, bottom panel). Overall, no clear trend was observed as would be suggested by the predictions [57,58], and the values for these parameters did not differ between fast and slow progressors.

### Co-receptor usage and cell tropism

Since co-receptor usage switches to an X4-utilization phenotype with disease progression in some adults and children, we evaluated co-receptor usage and phenotype of viral isolates from the two groups. We found that all viral isolates exclusively used CCR5 as a co-receptor (Table 1), exhibited macrophage-tropism, and did not infect T cell lines or form syncytia *in vitro*. The only exception was the 48-month isolate from infant 1690. For 1690, R5 co-receptor tropism was maintained until 42 months; after this time, the viral isolate exhibited dual X4/R5 co-receptor usage (Table 1), and infected both macrophages and MT-2 T lymphoblasts, where it formed syncytia (data not shown). To date, this is the only X4-utilizing virus isolated from our cohort, implying that while X4-utilizing subtype C HIV-1 can develop in patients, such development is uncommon and disease pathogenesis is not dependent on such phenotypic switches. To test whether the characterized subtype C Env sequences possessed co-receptor usage properties consistent with those defined for the virus isolated by co-culture, we generated Env chimeras by introducing the subtype C V1–V5 region into a subtype B NL4-3 Env expression vector. The chimeric Env constructs were then used to make pseudoviruses for evaluation of co-receptor usage in Ghost cell lines that express different co-receptors. All chimeras tested exhibited CCR5 tropism and lacked appreciable X4 tropism (data not shown). These findings are consistent with those obtained from experiments using primary isolates.

### Neutralization capacity of the baseline mother and infant plasma for the first infant viral isolate

Since maternal anti-HIV antibodies are transmitted from mother to infant, it is possible that they play a role in the selection of transmitted viruses and affect the disease course in the child. Therefore, we evaluated maternal and infant neutralizing antibody (Nab) titer at birth against the first infant viral isolate. The level of Nab was determined from the rapid (infants 1449, 2669 and 2873) and slow progressors (infants 1084 and 1984), as well as one slow progressor (infant 1157) described previously [33]. For rapid progressors, the first viral isolation was 2 months after birth, whereas the first viral isolates in the slow progressors are from 4 months (1084 infant) or 6 months (infant1157 and 1984). Our results (Table 2) indicate that the level of infant baseline Nab against infant first viral isolates was lower than the maternal baseline, implying that only a subset of the maternal neutralizing antibody was acquired by their infants. Comparison of the baseline Nab level between the corresponding mother and infant from each pair indicated that there is a direct correlation between the level of maternal Nab and the level of Nab passively transferred to their infants. Mothers with the low baseline Nab transferred the least Nab to their infants. But the level of Nab in either the maternal or the infant baseline plasma failed to differentiate rapid and slow progressors. For example, 88% neutralization by maternal baseline plasma was observed in one rapid (1449) and one slow (1157) progressor, respectively; whereas, in other cases, maternal baseline plasma from both rapid and slow progressors failed to effectively neutralize the earliest infant virus (infant 2873 vs.1984). Similarly, the neutralization capacity of the infants' plasma at birth against their first viral isolates does not differentiate the two groups. For example, both 2873 (rapid) and 1984 (slow) lack detectable Nab for their first viral isolates at birth.

**Table 2:** Neutralization activity (%) of baseline plasma for infant first viral isolates^1^

Patient number		Maternal plasma	Infant plasma
	1449	88	78
Rapid progressors	2669	93	45
	2873	42	0
			
	1084	79	60
Slow progressors	1157	88	69
	1984	55	0

^1^For rapid progressors, the first viral isolates were obtained at month 2 after birth, whereas the first viral isolates in the slow progressors were from month 4 (1084 infant) or 6 (infant 1157 and 1984). Neutralization assay was done using 1:20 of either maternal or infant plasma

### Longitudinal humoral immune responses of infected children

**Figure 4:**
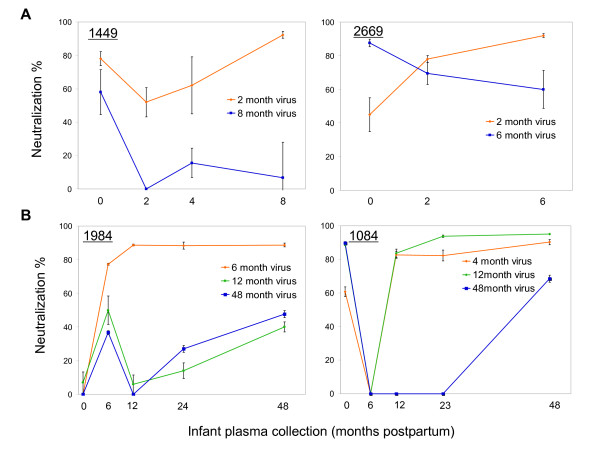
Contemporaneous and non-contemporaneous plasma neutralization activity against infant viral isolates was determined in TZM-bl cells. Panel A shows the results of the test plasma against infant 2 and 6 or 8-month viral isolates from two rapid progressors (1449 and 2669). Panel B shows the results of the test plasma against infant 4 or 6, 12 and 48-month viral isolates from two slow progressors (1984 and 1084). The test plasma was diluted to 1:20. Virus production in the supernatants was monitored by luciferase activity at 2 days post infection. Luciferase activity in the control wells containing no plasma was defined as 100%, and the neutralization capacity of the test plasma was calculated relative to this value.

To further characterize the infant antibody responses, we quantified neutralization by autologous sera from various timepoints for the first and last viral isolates from both groups. The neutralization profiles of two representatives from each group are shown in Figure 4. For the rapid progressors (1449 and 2669), we observed variability in the baseline neutralizing antibody activities acquired from the mother (Figure 4A). In 1449, the initial activity against the earliest virus (78% neutralization) declined, prior to the initiation of a *de novo *infant humoral immune response near the time of the first virus isolation, which rose thereafter. Whereas, in 2669, the maternal transfer was less effective (only 45 % neutralization), but the infant *de novo *neutralizing response was evident by two months since the neutralization was higher than baseline. The *de novo *development and maintenance of effective neutralization against the 2-month viral isolates appeared early in both cases and increased in activity until the end of follow-up (Figure 4A). In contrast, neutralizing antibodies against the late viral isolates (8 months for 1449; 6 months for 2669) were lower in magnitude and decreased throughout the disease course (Figure 4A).

Similarly, the autologous plasma neutralization of slow progressor infant early (4 or 6-month) and late (48-month) viral isolates was evaluated, and two representatives (1984 and 1084) are shown in Figure 4B. In the slow progressor 1084, a substantial amount of Nab was detected at birth, but decayed to zero by six months. Subsequently, the child developed an effective neutralizing response against both the earliest virus and the contemporaneous (12 and 48-month) viruses. In contrast, slow progressor 1984 received no detectable Nab from the mother, but mounted an effective neutralizing response by 6 months whose magnitude was directly correlated with the timepoint of virus isolation, with 6-month virus being more effectively neutralized than 12 and 48-month viruses. It is apparent that in slow progressors there are infants who passively acquired neutralizing activity (1084), while others (1984) did not. Therefore, it is unlikely that rapid progression is due to receipt of lower maternal Nab, or that slow progression is due to acquisition of high level of maternal Nab or the development of a higher or more durable *de novo *humoral response.

### Replication of viral isolates from both rapid and slow progressors

**Figure 5:**
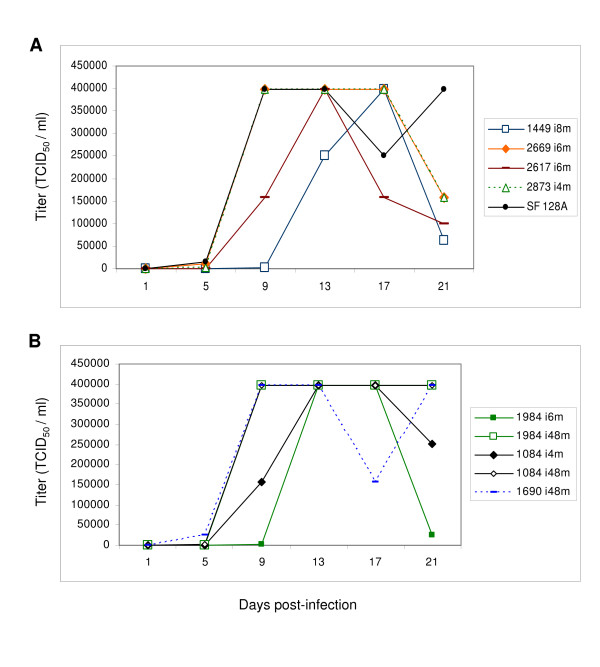
Replication of viral isolates from rapid and slow progressors in PBMC. Panel A shows the replication properties of the last viral isolates (4-month for 2873, 6-month for 2617 and 2669, 8-month for 1449) from four rapid progressors. Panel B shows the replication properties of the first (6-month for 1984, 4-month for 1084) and last viral isolates (48-month for 1984, 1084 and 1690) from slow progressors. The laboratory viral strain SF 128A was used as control. Each 2000 TCID_50 _viral inoculum was added to 2 × 10^7 ^PHA stimulated PBMC from a pool of two HIV-1 seronegative blood donors. Virus titer (TCID_50_/ml) was measured by infections of TZM-bl cells by viruses harvested from days 1, 5, 9, 13, 17 and 21.

In order to determine whether there are differences in the rates of replication among the viral isolates from rapid and slow progressors, the replication of the first viral isolates (slow progressor only) and last viral isolates (all 7 infected children) in PBMC was determined (Figure 5). The titer (TCID_50_/ml) of the last viral isolates from all rapid progressors (4, 6 or 8-month after birth) displayed steady increase after 5 or 9 days incubation and peaked by 9 (infant 2669 and 2873), 13 (infant 2617) or 17 (infant 1449) days (Figure 5A). For slow progressors, the first viral isolates (6-month for 1984, 4-month for 1084) displayed similar replication kinetics compared to the rapid progressors. However, when comparing the first and last viral isolates from the slow progressors, the late viruses (48-month for infants1984, 1084 and 1690) showed a slightly more rapid replication kinetics than the early viruses, with a peak value by 13 days, while the late viruses peaked by 9 days (Figure 5B).

## Discussion

Longitudinal changes in viral genetic variation, immune responses, and disease progression have rarely been investigated in HIV-1 subtype C infected children. We have previously characterized the evolution of the Env C2-V4 region of subtype C HIV-1 and the humoral immune response from one infected infant. In the present study, we expanded our study by correlating the changes of the Env longitudinally with disease outcome, in seven children, divided into two groups based on rapid or slow disease progression. In addition, with these two groups, we were able to examine the contribution of Env length and glycosylation in disease progression, and the role of humoral immunity, both passively acquired and developed *de novo*, to clinical outcomes.

Phylogenetic analyses show that maternal and infant viruses were epidemiologically linked in each of the seven pairs, and support the concept that selective transmission occurred [33,59-61]. Rapid progressors, those who died in the first 12 months, received and maintained a genetically homogeneous viral population throughout the short disease course. Slow progressors initially also exhibited low levels of variation, but attained higher levels of diversity over time. These findings are consistent with previous studies that showed higher genetic diversity associated with slow disease progression in children [33,53,54].

In both groups of children, a large number of unique, but closely related haplotypes were sampled, matching predictions for a population that was exponentially growing in size from a homogeneous starting point. Estimates of dN/dS can be used to determine whether selective pressure, in addition to expanding population size, played a role in the diversification of the infant viral populations. Our data show that dN/dS values were high in all 7 individuals, exceeding 1.0 in 7 of 24 populations sampled. Values of dN/dS greater than 1.0 provide evidence of positive Dawinian selection [62].

One of the primary selective pressures acting on Env is neutralizing antibody. The earliest infant Nab responses are largely due to passive transfer from the mother. Passively acquired maternal immunity can play a critical role in protecting infants from infections; however, the specific contribution of maternal or passively-acquired neutralizing antibodies in limiting HIV-1 transmission or disease progression in children is not well understood. Our observations indicate that the neutralizing activity in maternal and infant baseline plasma varied in its effectiveness for the initial infant virus but did not differentiate rapid from slow progressors. Since our assays for Nab activity relied on co-cultured virus, and selection during co-culture may bias the results away from the main phenotype of virus in the original population, the lack of difference between groups should be taken as a tentative result. Nevertheless, consistent with other findings [33,63,64], all children developed *de novo *neutralizing responses within the first 6 months post-infection regardless of the disease course. But our results show that even when children develop effective *de novo *neutralization responses, they may still progress rapidly (Figure 4A). In contrast, we also observed children who failed to mount high neutralizing responses to later virus, yet have remained clinically asymptomatic throughout the study (Figure 4B). These findings indicate that the development of effective neutralizing responses in children fails to protect them from disease progression, but surprisingly, failure to develop effective responses is not predictive of rapid progression. Moreover, there is no association between the replication kinetics and disease progression, since viral isolates isolated from similar time points (4–8 month) from both rapid and slow progressors replicated with similar pattern (Figure 5A and 5B), even though the late viruses from slow progressors replicated slightly faster than early viruses from the same hosts (Figure 5B). Similarly, our study did not reveal any differences in cytopathicity of the viruses from either progressors or non-progressors from different time points, suggesting a lack of correlation between viral cytopathicity and disease progression among the viruses that were analyzed.

The genotypic and phenotypic parameters leading to preferential transmission of particular virus variants from donor to recipient remain unclear. In heterosexual transmission between discordant couples, it was found that subtype C viruses with shorter V1–V4 regions and fewer putative glycans were preferentially transmitted and were neutralization sensitive [57,58]. In addition, another study of heterosexually acquired subtype A viruses suggested that transmitted viruses have shorter V1–V2 length and few N-linked glycosylation sites [65]. An extension of these findings is that evolution in the newly infected individual would lead to longer and more glycosylated Env proteins with time. These patterns have not been confirmed in subtype B sexual transmission [65-67]. The genotypic and phenotypic parameters leading to preferential transmission of particular virus variants were also evaluated in mother to child transmission. An investigation of subtype A mother to child transmission has revealed that the transmitted viruses were more resistant to neutralization by maternal plasma although the viruses harbored fewer putative glycosylation sites [64]. In our study, we have observed that both neutralization sensitive and resistant viruses were transmitted to both slow and rapid progressors. It is worth noting that contrasting results between sexual transmission and vertical transmission studies could be due to fundamental differences between these processes, since vertical transmission occurs in the presence of neutralizing antibodies, but in sexual transmission there are presumed to be no baseline antibodies present.

It has been hypothesized that the extensive glycosylation of the HIV-1 Env shields the protein from immunological recognition, or conversely, targets recognition to less functionally constrained domains where hypervariability can be tolerated [68]. Interestingly, neither pattern was confirmed with later viruses in our infant samples, suggesting that lengthening of the V1–V5 domain and acquisition of glycosylation sites were not always a component of glycoprotein evolution in newly infected individuals (Figure 3 and Table 1). Only in one case (infant 1084), a pattern consistent with this hypothesis was obtained, with increasing V1–V5 length and number of PNGS (Figures 3). Collectively, our results highlight the necessity to refine our understanding of the relationships between viral genotype, viral phenotype and different routes of transmission. Our observations and those of others also stress the need to further explore genetic and immunologic correlates of mother to child transmission in non-B subtypes.

Comparison of the rates of non-synonymous and synonymous substitutions has been used as an index of selective pressure exerted by the immune system [20,55,69]. There are reports that higher dN/dS ratios are linked with long-term survival [20,55]; however, we found that the highest dN/dS value was estimated for envelopes from a rapid progressor child at the final timepoint prior to death (Table 1, 8-month sample from infant 1449). In addition, dN/dS values were highly variable in both groups and not statistically different. Despite the variation in dN/dS values, the estimates were high in all cases, suggesting that natural selection is a strong determinant of the diversification and evolution in the Env glycoprotein. Further evidence of this selective pressure comes from the observation that amino acid replacements are not evenly distributed in the protein sequence, but occur in 'hot-spots' in particular domains (Figure 2). We can predict two broad mechanistic explanations for these changes; (1) they modulate glycoprotein function thus enhancing viral fitness (currently under investigation), (2) they modulate immune recognition of the viral glycoprotein by altering epitopes. Despite differences in timing of sampling, or in ultimate disease outcome, some hot spots are shared among all children, and no hot spot differentiates the rapid from the slow progressors. One example of these common hot-spots is the region in C3 just carboxy-terminal to the V3 loop. Structurally this domain corresponds to alpha helix 2 from the alignment of HXBC2 to the intact SIV atomic structure [56]. This sequence, which is perpetually changing, is located on the silent face of the trimeric structure as determined for subtype B. The clustering of polymorphisms as well as the differential binding of antibodies from subtype B versus C infected individuals to this region points to this as a good candidate for a subtype C neutralizing epitope.

Genetic assessment of variation, as indicated by dN/dS, shows that the protein is undergoing selective changes in a non-random fashion. Nevertheless, the magnitude and distribution of these changes do not segregate slow and rapid progressors. In addition, the apparent clustering of positively selected residues in particular regions of the subtype C glycoprotein that are normally not subject to high levels of mutation in subtype B implies significant structural differences between the glycoprotein of the two subtypes.

The sole parameter that discriminates rapid and slow progressors appears to be time to death. We found that neither genetic variation in *env*, nor maternal neutralizing activity at parturition, nor the level of passively acquired neutralizing antibody, nor the level of the *de novo *neutralization response was linked to differences in disease progression in the children studied. However, characteristics of the transmitted viruses other than Nab and the genetic make up of viral populations could be linked to disease progression. Such factors might include, but are not limited to, the fitness of the transmitted viruses, binding affinity to CD4 and/or co-receptors, and cell mediated immunity. The potential role of these factors in predicting disease outcome in subtype C HIV-1 infected children will be the focus of further investigations.

## Conclusion

In this study, we examined the evolution of Env V1–V5 region from seven subtype C HIV-1 perinatally infected children with different clinical outcomes. In addition, the humoral immune responses of the infected children during disease progression were also evaluated. Our findings demonstrate that neither the level of maternal baseline neutralizing antibody nor the portion passively transferred to the infant is predictive of disease outcome. Inspection of the neutralization susceptibility to maternal baseline plasma indicated that both neutralization sensitive and resistant viruses were transmitted to the infected children with different disease courses. Moreover, we showed that the development of effective *de novo *neutralization response in infected children did not differentiate rapid from slow progressors. Genetic analysis of Env V1–V5 region could not segregate rapid and slow progressors, suggesting that time to death may be the parameter that discriminates rapid and slow progressors.

## Patients and methods

### Patient population and sample collection

Seven mother/infants pairs 1449, 2669, 2873, 2617, 1984, 1084 and 1690 were recruited into the study. Venous blood was obtained from the mother before delivery and from the infant within 24 hours of birth. Follow-up blood specimens were obtained at 2, 4, and 6 or 8 months (for rapid progressors) and at regular intervals through 48 months (for slow progressors). The baseline HIV-1 serological status of the mother was determined by two rapid assays, Capillus (Cambridge Biotech, Ireland) and Determine (Abbott laboratories, USA). Positive serological results were confirmed by immunofluorescence assay (IFA), as previously described [70]. HIV-1 infection in the infants was detected by sequential viral isolation from the infants' peripheral blood mononuclear cells (PBMC), as previously described [33], and by PCR of the HIV-1 provirus *env *gene from genomic DNA. The first timepoint for positive PCR from each infant is indicated in Table 1.

### Cell tropism and chemokine co-receptor usage

The syncytium-inducing (SI) or non-syncytium-inducing (NSI) phenotype was determined by infecting MT-2 cells as described [33]. Virus was scored as 'SI' if syncytia and increasing level of p24 antigen were observed within a 10-day period and as 'NSI' if syncytia failed to form within that time. To further define the viral tropism, viral replication was assessed in primary monocyte-derived macrophages (MDM) and in the MT-2 T-cell line, as described [33].

Co-receptor usage was defined using Ghost cell lines that express specific co-receptors (Ghost-CXCR4 cells [CXCR4], Ghost-CCR5 cells [CCR5] and Ghost-CCR3 cells [CCR3])(NIH AIDS Research and Reference Reagent Program, Division of AIDS, NIAID, NIH from Dr. Vineet KewalRamani and Dr. Dan Littman) with standard procedures as recommended by the supplier. Uninfected control produced only one to two GFP-expressing cells per well. HIV-1 strains NL4-3 and SF128A were used as positive controls for CXCR4 and CCR5 utilization, respectively.

### Virus neutralization assay

Virus sensitivity to neutralization by maternal or infant plasma-derived antibody (1:20 dilution of plasma) was determined by infections of TZM-bl cells (NIH AIDS Research and Reference Reagent Program catalog no. 8129, TZM-bl) as described [33].

### Viral replication assay

Two thousand TCID_50 _(Tissue Culture Infectious Dose) of the viral isolates from early or late time points were added to a T-25 flask containing 2 × 10^7 ^PHA-stimulated PBMC from a pool of two HIV-1 seronegative blood donors. The subtype B laboratory isolate SF 128A [71] was used as a control. After incubation at 37°C for 6 hours, cells were washed 3 times with PBS and replenished with fresh medium. All infected cultures were sampled and supplemented with fresh culture medium at days 1, 5, 9, 13, 17 and 21-postinfection. Virus titer (TCID_50_/ml) was measured by infection of TZM-bl cells (NIH AIDS Research and Reference Reagent Program catalog no. 8129, TZM-bl).

### Polymerase chain reaction, gene cloning, sequencing and subtype identification

Genomic DNA was purified from uncultured patient PBMC using the Puregene kit (Gentra Systems). Primers used for amplification of the subtype C *env *gene were designed based on a reference alignment of all HIV-1 subtypes obtained from the Los Alamos Sequence Database. Nested PCR was used to amplify a 1100 bp fragment spanning the V1–V5 region of the *env *gene from samples collected at birth through 48 months. In order to minimize the PCR bias, Env V1–V5 region were amplified in duplicates in the first-round PCR of each maternal and infant sample. First-round PCR products were combined and used as template in a second-round PCR. Primers used in the first round PCR were: forward primer EnvF1: 5'-GATGCATGAGGATATAATCAGTTTATGGGA (corresponding to position 6533 to 6562 of HIV-1 HXB2 strain), reverse primer EnvR1: 5'-ATTGATGCT GCGCCCATAGTGCT (position 7828 – 7806). Internal primers used in the second round PCR were EnvF2: 5'-AGTTTATGGGACCAAAGCCTAAAGCCATGT (position 6552 to 6581) and EnvR2: 5'-ACTGCTCTTTTTTCTCTCTCCACCACTCT (position 7762 to 7734). First and second round PCR were carried out at 94°C for 2 min; 94°C for 30 sec, 55°C for 1 min, 68°C for 1 min for 35 cycles; 68°C for 7 min using a Perkin-Elmer thermocycler. Amplified fragments were cloned into the pGEM-T Easy vector (Promega) and sequenced in both directions with dideoxy terminators (ABI BigDye Kit). The V1–V5 region of the *env *gene was analyzed.

### Sequence analyses

Subtype affiliation was determined by comparing patient *env *sequences to a reference panel from the HIV database. Maximum likelihood (ML), Minimum-Evolution (ME) and neighbor-joining (NJ) phylogenetic analyses were done to visualize how viral populations within patients change with time. Maximum likelihood searches were conducted in Treefinder [72] using an independent model of nucleotide substitution for each codon position (GTR+Γ). Minimum-Evolution and Neighbor-joining analyses were performed in MEGA [73] using the Kimura 2 parameter nucleotide substitution model to estimate genetic distances. Support for the nodes in ME and NJ was evaluated by running 1000 bootstrap pseudoreplicates.

For each infant, samples were grouped according to the time of collection, and analyzed independently. To keep a consistent set of sites in the analyses, positions where putative insertions or deletions were inserted for alignment purposes in any timepoint were excluded from population level comparisons. Variation in the pattern of genetic diversity for each time-point was explored using MEGA3.1 [73], DNAsp ver 4.10 [74] and Datamonkey [75]. The number and location of putative N-linked glycosylation sites (PNGS) was estimated using N-GlycoSite from the Los Alamos National Laboratory database. Within each infant, temporal changes in viral diversity and viral divergence from the earliest population were calculated. Viral diversity estimates correlate with the effective population size of viral populations and were estimated based on the average number nucleotide differences between sequences, whereas viral divergence was calculated as the average genetic distance relative to the earliest population collected.

The overall dN/dS (non-synonymous changes relative to synonymous changes) values for each contemporaneous data set were calculated using Datamonkey [75], and used to estimate the relative strength of natural selection in each set of sequences. In addition, the number of synonymous and non-synonymous substitutions observed per site in infant Env (V1–V5) domains was calculated by Datamonkey, and the distribution of non-synonymous substitutions along the sequence was plotted to identify regions that accumulate most variations ('hot spots'). These regions were mapped on the structural predictions from Chen et al. [56] using their alignment of the SIV Env structure with HIV.

## Authors' contributions

HZ carried out the PCR, cloning, and sequencing. FH, GO, HZ and XH performed the sequencing analysis by computer program. JH carried out viral isolation, viral tropism, co-receptor usage and neutralization assay. CK was involved in patient recruitment and follow-up. RR contributed to experimental design. HZ, FH, JW, CM, GO, and CW participated in the experimental design, data interpretation and writing of the manuscript.
